# Sociodemographic and clinical profile of immigrants hospitalized in psychiatric facilities in Tunisia

**DOI:** 10.1192/j.eurpsy.2024.1277

**Published:** 2024-08-27

**Authors:** F. Tabib, S. Omri, R. Bouaziz, R. Feki, I. Gassara, L. Zouari, J. Ben Thabet, N. Charfi, M. Maalej, N. Smaoui, M. M. Bouali

**Affiliations:** Department of Psychiatry C, UHC Hedi Chaker, Sfax, Tunisia

## Abstract

**Introduction:**

In an increasingly interconnected world, migration has become a defining characteristic of the 21st century. While immigration offers new beginnings and prospects, it also presents unique challenges, particularly concerning mental health.

The experience of migrating can exert pressure on mental health through factors such as acculturation stress, discrimination, and economic hardships. These challenges can, in turn, contribute to the development of mental health issues.

**Objectives:**

To study the socio-demographic and clinical profile of immigrants hospitalized in the “C” psychiatry department, Hedi Chaker Hospital, in Sfax, Tunisia.

**Methods:**

We conducted a retrospective descriptive study of immigrants hospitalized in the psychiatry department “C”, Hedi Chaker Hospital, Sfax Tunisia from 2011 to 2023. Socioeconomic data and clinical profiles of immigrants were collected from archived files.

**Results:**

The total number of immigrants hospitalized during these 12 years was 32, with an average age of 28.81 years ±7.8 years, all of them were males, as the psychiatric department “C” only hospitalizes men.

All were of African origin, of whom 21.9% (n=7) had Libyan nationality, 15.6% (n=5) had Somali nationality and 12.5% (n=4) had Sudanese nationality. Communication with them was possible in 87.5% of cases, primarily through the native Arabic language in 56.3% of instances. Illegal immigration was the most prevalent form, accounting for 75% of cases. During the immigration process, 18.8% of individuals reported experiencing violence.”

The majority of hospitalized immigrants were single (71.9%), had a primary school education (37.5%), a low socio-economic level (81.3%), and no profession (59.4%). 21.9% of them had received social assistance, and 59.4% lived in a refugee camp. Psychoactive substance consumption was reported by 53.1% of our study population.

Regarding the clinical profile of the population, 21.9% had a history of somatic conditions, 43.8% had a psychiatric history, including 9.4% who had attempted suicide, and 34.4% who had experienced traumatic events since arriving in Tunisia. The primary reason for hospitalization was behavioral disorders in 71.9% of cases and suicide attempts in 15.6%. The most prevalent diagnoses were schizophrenia (50%), and bipolar disorder (18.8%). Upon discharge, 15.6% encountered administrative issues.

**Conclusions:**

Hospitalized immigrants exhibit diverse socio-demographic and clinical profiles. These findings underscore the significance of acquiring a deeper understanding of the mental health needs and existing barriers to healthcare within various immigrant communities. This is particularly crucial as immigration continues to be a central focus in Tunisia’s public policies and discussions.

**Disclosure of Interest:**

None Declared

